# Fellowships, Grants, & Awards

**Published:** 2005-01

## Metals in Medicine

The objective of this program announcement (PA) is to encourage research that bridges the areas of inorganic chemistry and medicine in continuation of PA-01-071. The National Institute of General Medical Science (NIGMS) is joined in this announcement by the NIEHS and the NIH Office of Dietary Supplements (ODS).

The mechanisms by which organisms control transition metal ions and the roles of these metals in cellular regulation and signaling in health and disease are of principal interest. The interactions of synthetic inorganic complexes with living systems and their components are an additional area of interest. These areas are linked by the need to involve researchers having a deep understanding of inorganic chemistry in medically relevant research. Much of the work is expected to involve collaborations including chemists, biologists, and medical researchers. The results will be relevant to understanding the mechanisms of metal handling by biological systems and the basic cellular roles underlying the nutritional requirement for essential metals. It is expected that this research will also contribute to the identification of new targets for drug discovery, diagnostics, and future therapeutic approaches involving metal complexes, although drug development, per se, is not a focus of the program.

A higher-order problem presents itself in understanding how the genome-encoded components and the other molecules are constituted in networks of interacting molecules with particular distributions in time and space. Advances in imaging techniques and analytic methods are beginning to yield copious quantitative and spatial data on specific molecules in biological systems. Knowledge of the network and changes in its components over time, and the local rules by which the individual components distribute material and information, will substantially advance our knowledge.

Studies of metalloenzyme structure and function, mechanisms of action, and inhibition are currently well supported and produce results that are utilized in the design of new diagnostic and therapeutic products. Additional stimulation of this area is not needed. In contrast, work in other areas of bioinorganic chemistry lags behind its potential application to human health. These areas include 1) mechanisms of metal metabolism as well as the roles of metals in regulation of cell function and cell–cell interaction, and 2) basic research toward diagnostic and therapeutic applications of metal complexes and of metal chelators and toward exploiting the unique properties of metals for therapeutic applications. The emphasis of this announcement is on the ions, complexes, and organometallic compounds of the transition metals known as lanthanides and actinides, post-transition metals, and metalloid elements.

### Metal Metabolism and Regulation.

Metal metabolism is emerging as an exciting area of cell biology and a potential area for therapeutic intervention. Normal metal metabolism appears to maintain free metal ion concentrations at a very low level and to deliver metals very selectively to their sites of action, while maintaining tight control over their reactivity. Aberrant metal metabolism contributes to pathological conditions such as Menkes’ disease, Wilson’s disease, and hemochromatosis. Intercepting normal metalation reactions may be a way to control metalloprotein activity. Metals may also be associated with the pathology of protein aggregates such as those formed by prions and in Alzheimer’s disease. Metals have also emerged as important sensors and transducers of information with roles in regulation and neurotransmission.

Areas of interest include 1) improved metal ion sensors to study cellular metal ion concentrations and localization; 2) reagents suitable to manipulate those concentrations; 3) identification and characterization of the macromolecular players and vesicular compartments involved in metal ion homeostasis and metal trafficking; 4) elucidation of the roles of metals in cell regulation, signal transduction, and cell–cell signaling; 5) identification and understanding of mRNAs and metal-, oxygen-, and redox-responsive transcriptional and translational regulators, and their potential as therapeutic targets; 6) elucidation of the mechanistic roles of essential trace elements for which metabolic functions are not yet clearly established; 7) analytical tools that accurately monitor biologically important pools, storage pools, and the chemical speciation of metals; 8) biomarkers of exposure and mechanisms of metal toxicity; 9) biomarkers for variable susceptibility to metal toxicity in the human population; and 10) chelation chemistry that can serve as the foundation for therapies to ameliorate aberrant metal accumulations and the effects of toxic exposures.

### Interactions of Metal Complexes with Living Systems.

The therapeutic application of metal complexes is an underdeveloped area of research. Basic principles to guide the development of metallopharmaceuticals are lacking. Metal-containing agents may offer unique therapeutic opportunities. However, significant obstacles, including potential metal accumulations and toxicities, require further research before the promise of medicinal inorganic chemistry can be realized.

Metal complexes may be useful as research probes of biological function, as intermediary lead compounds in the development of non–metal-containing therapeutics, and as potential diagnostic and therapeutic agents. Opportunities exist to exploit the unique properties of metal complexes, (e.g., hydrolytic and redox activity, Lewis acidity, electrophilicity, valency, geometry, magnetic, spectroscopic, radiochemical properties) to measure and/or alter cellular functions. The actions of these compounds may provide insights that are different from those that can be achieved through other chemical, biochemical, or genetic manipulations. Similarly, the actions of metal complexes in whole living organisms are expected to differ in general from the actions of non–metal-containing agents and may offer unique research, diagnostic, or therapeutic opportunities. Principles are needed for the design of safe metal-containing therapeutics.

Another goal of this program is to utilize the power of inorganic chemistry to provide new knowledge of and new approaches for intervention in biological systems. Still another goal is to improve understanding of the reactions of metal complexes in living systems to improve the specificity of these interactions and gain control over the potential toxicity of synthetic metal complexes. The long-term goal is to establish the basic principles of an inorganic medicinal chemistry that will allow for rational design and screening of potential metallopharmaceuticals in the future.

Areas of interest include 1) reactions of metal complexes with cellular constituents (e.g., DNA, RNA, proteins, lipids, carbohydrates, redox substrates, signaling molecules); 2) reactions of metal complexes within the cellular milieu and *in vivo*; 3) uptake of metal complexes into cells and delivery to specific cellular compartments; 4) interactions of metal complexes with specific enzymes and receptors; 5) mechanisms by which synthetic metal complexes recruit cell cycle, signal transduction, and other metabolic pathways to alter cell functions; and 6) structure–activity relationships for ligand design to control metal complex activity and stability *in vitro* and *in vivo*.

The NIH Metals in Medicine meeting report includes a list of specific research opportunities and challenges. This list is intended to be illustrative, not exhaustive. Investigator-initiated ideas are welcome on any subject that will contribute to the objectives listed in this PA.

Research encouraged by this announcement may utilize any appropriate experimental organisms or model systems. For some problems, interesting discoveries may be found in microorganisms from unusual environments and atypical experimental organisms. For other problems, yeast, common invertebrate and vertebrate model organisms, and human cell/tissue cultures may be appropriate. Investigators considering human clinical trials are strongly encouraged to contact the program staff.

This funding opportunity will use the regular research (R01), exploratory research (R21), and program project (P01) award mechanisms. For a description of the R21 grant mechanism see http://grants.nih.gov/grants/funding/r21.htm. For descriptions of the P01 grant mechanism see http://www.nigms.nih.gov/funding/grntmech.html#b (NIGMS) and http://www.niehs.nih.gov/dert/programs/p01.htm (NIEHS).

This funding opportunity uses just-in-time concepts. It also uses the modular as well as the nonmodular budget formats (see http://grants.nih.gov/grants/funding/modular/modular.htm). Specifically, if you are submitting an application with direct costs in each year of $250,000 or less, use the modular budget format described in the PHS 398 application instructions, available at http://grants1.nih.gov/grants/funding/phs398/phs398.html in an interactive format. Otherwise, follow the instructions for nonmodular research grant applications. For further assistance, contact GrantsInfo at 301-435-0714 (telecommunications for the hearing impaired: TTY 301-451-0088) or by e-mail: GrantsInfo@nih.gov.

Applications must be prepared using the PHS 398 application instructions and forms (rev. 5/2001). Applications must have a Dun & Bradstreet (D&B) Data Universal Numbering System number as the universal identifier when applying for federal grants or cooperative agreements. This number can be obtained by calling 1-866-705-5711 or online at http://www.dnb.com/us/. The D&B number should be entered on line 11 of the face page of the PHS 398 form. Applications must be submitted on or before the receipt date described at http://grants.nih.gov/grants/funding/submissionschedule.htm. The complete version of this PA is available at http://grants.nih.gov/grants/guide/pa-files/PA-05-001.html.

Contact: Peter C. Preusch, Division of Pharmacology, Physiology, and Biological Chemistry, NIGMS, Bldg 45, Rm 2AS.43C, MSC 6200, Bethesda, MD 20892-6200 USA, 301-594-5938, fax: 301-480-2802, e-mail: preuschp@nigms.nih.gov; Claudia Thompson, NIEHS, DERT/OPD/CEMBB, MD EC-21, PO Box 12233, Research Triangle Park, NC 27709 USA, 919-541-4638, fax: 919-541-4606, e-mail: thompso1@niehs.nih.gov; Becky Costello, ODS, NIH, Bldg 31, Rm 1B25, MSC 2086, Bethesda, MD 20892-2086 USA, 301-435-2920, fax: 301-480-1845, e-mail: costellb@od.nih.gov. Reference: PA No. PA-05-001

## NIGMS National Centers for Systems Biology

The National Institute of General Medical Sciences (NIGMS) currently supports the analysis of complex biological systems through investigator-initiated research project grants. The resources needed to conduct the multifaceted, multidisciplinary projects that may be required to achieve significant advances in these complex areas may be beyond the scope of the typical R01 or P01 grant. Therefore, this request for applications (RFA) presents an opportunity for applicants to assemble large teams of investigators from diverse disciplines that may not be possible with other funding mechanisms.

The biomedical sciences have undergone a fundamental shift in the conceptual and technical approaches that can be applied to certain problems of profound importance. These problems center on understanding the behavior of biological systems whose function is the product of spatial and temporal ordering of myriad interacting components. Modeling approaches are being used to understand the orderly development of biological pattern in organisms such as *Drosophila* and *Caenorhabditis elegans*, and at the clinical level, new approaches are being explored to understand the integrated activity of tissues and organs.

Part of the impetus for systems-scale approaches rests on advances in acquiring data of the necessary quality and quantity to permit computational modeling. Among the most striking examples are the availability of complete DNA sequences for hundreds of organisms, including humans, and the availability of high-throughput instrumentation for analyses of gene function such as gene expression microarrays and proteomics technologies. These advances have made it feasible to generate a truly comprehensive parts list for any organism and to track changes over time. Ultimately, it should be possible to enumerate all the informational units of the genomes (protein coding genes, non–protein coding genes, regulatory regions), their processed forms, and their dynamic presence in cells.

Rapid advances in large-scale data collection and analysis have given scientists a global yet detailed view of cellular processes, instead of focusing on individual molecules or a small number of interacting molecules. Unprecedented opportunities have emerged that may open the door to uncover hidden rules governing the ensemble of biomolecules working concertedly to perform certain functions in the cell. In the meantime, substantial challenges in information integration, interpretation, and representation have arisen. In order to move beyond the phase of cataloguing the parts list and truly transform data into knowledge, and knowledge into principles, iterative cycles of data collection and model generation and validation will be necessary.

A higher-order problem presents itself in understanding how the genome-encoded components and the other molecules (metabolites, ions, water, etc.) are constituted in networks of interacting molecules with particular distributions in time and space. Advances in imaging techniques and analytic methods are beginning to yield copious quantitative and spatial data on specific molecules in biological systems. Knowledge of the network and changes in its components over time, and the local rules by which the individual components distribute material and information, will substantially advance our knowledge.

At the organism level, phenotype must take into account the relationships and interactions of biological and environmental variables. Basic biological systems—including gene sequences, structures, and pathways that direct metabolism and development—vary within individuals, among individuals, among populations, and among species. Advances in complex systems-level understanding must ultimately include models that account for these variations.

Medical, biotechnological, and other uses of biological information increasingly depend on our ability to understand the principles and dynamics that explain the behavior of the system as a whole. Whether the goal is to understand the consequences of disease or injury, identify particular molecular targets for drug interventions, or modify the metabolism of microorganisms to produce medicines, the challenge is predictability. Predicting how the system of interest will respond to an intervention is a computational problem. For biological systems, this challenge is daunting.

Parallel to scientific challenges are organizational and educational challenges. At the institutional level, building cohesive multidisciplinary research teams by integrating expertise across traditional disciplinary boundaries is not a simple undertaking. Beyond institutions, excessive overlap and redundancy in project selection and tool development exists in the research communities that could be reduced by promoting communications, collaborations, and technology and data sharing. The emergence of new science demands an adequate workforce of new scientists. Training for the future leaders of systems biology research who are knowledgeable and skilled in both experimental and computational subjects is timely. Good mechanisms and plans to address these challenges are significant tasks of the centers.

High priority will be given to projects that integrate multi-investigator, multidisciplinary approaches with a high degree of interplay between computational and experimental approaches. Innovation is critical for both research project design and infrastructure design with a mission of serving communities beyond the participating investigators, institutions, and collaborators. A variety of organizational models are possible; it is not the intent of this RFA to prescribe any particular one.

The NIGMS awarded two centers under this program in 2002 (http://www.nigms.nih.gov/news/releases/complex_centers.html), two centers in 2003 (http://www.nigms.nih.gov/news/releases/complex_centers-2003.html), and one center in 2004 (http://www.nigms.nih.gov/news/releases/quantitative_bio_center.html). Potential applicants should become familiar with the research focuses of the existing centers. Research conducted by the future centers should complement and enhance projects already funded.

Some groups interested in the subject of this RFA might find the P01 mechanism more suited to the scale of their efforts; they should consult the prior announcement at http://grants.nih.gov/grants/guide/pa-files/PA-98-077.html.

The NIGMS intends to support systems biology research for the areas that are central to its mission of supporting basic biomedical research, and that focus on developing new computational approaches to biomedical complexity. Research areas that historically have been computationally based (e.g., molecular structure and modeling) are excluded as a focus of this center program. Research focusing on disease processes and their specific organ systems is not eligible. NIGMS mission areas include, but are not limited to, the following: 1) signaling networks and the regulatory dynamics of cellular processes such as cell cycle control, transient complex formation, organelle biogenesis, and intercellular communications; 2) supramolecular machines, such as the replisome, spliceosome, and molecular motor assemblies in cell division and motility; 3) pattern formation and developmental processes in model systems (e.g., *Drosophila*, *C. elegans*, etc.); 4) metabolic networks and the control of the flux of substrates, intermediates, and products in cell physiology; 5) organ system networks involved in multiorgan failure in shock, trauma, and burn injury; and 6) genetic architecture of biological complexity related to inherited variation and environmental fluctuations.

The NIGMS National Centers for Systems Biology will be expected to provide national leadership in systems biology research and training. To do so, they will be expected to support training and outreach activities that will ensure the flow of information and expertise both into and out of the centers. Centers should have plans to bring the most advanced technologies developed at other laboratories to the centers and to disseminate expertise and knowledge to a wider community through collaborations, visiting investigatorships, fellowships, center websites, workshops, symposia, summer courses/internships, and/or other means. To maximize the impact, centers should conduct training at multiple levels appropriate to their institutions. Incorporation of developmental research projects led by junior and new investigators into the center research and development plans is strongly encouraged. Over a period of time, centers should evolve into integrated research, training, and knowledge exchange headquarters of scientific communities that will be the engines for coordinated scientific discoveries. The centers should also have plans for outreach to undergraduate institutions, including minority-serving institutions. Information on relevant minority-serving institutions may be obtained by consultation with staff of the NIGMS Division of Minority Opportunities in Research (http://www.nigms.nih.gov/about_nigms/more.html).

In addition to research and training contributions, successful centers will provide their home institutions with the means to implement organizational and professional changes that will make systems biology research an attractive career option for both established and entry-level investigators.

This funding opportunity will use the NIH P50 Research Center Grant award mechanism. As an applicant, you will be solely responsible for planning, directing, and executing the proposed project. This RFA is a one-time solicitation and may be reannounced in the future. The earliest expected award date is in December 2005. Applications that were submitted in response to previous RFAs of this program but unfunded may be revised and resubmitted for this RFA.

The NIGMS intends to commit up to $7 million in fiscal year 2006 to fund one to three new P50 center grants in response to this RFA. An applicant may request a project period of up to five years and a budget for direct costs of up to $2 million per year, exclusive of subproject fiscal and administrative costs (see http://grants.nih.gov/grants/guide/notice-files/NOT-OD-04-040.html).

The PHS 398 application instructions are available at http://grants.nih.gov/grants/funding/phs398/phs398.html in an interactive format. For further assistance, contact GrantsInfo by calling 301-435-0714 or e-mailing GrantsInfo@nih.gov.

Applications must be prepared using the PHS 398 application instructions and forms (rev. 5/2001). Applications must have a Dun & Bradstreet (D&B) Data Universal Numbering System number as the universal identifier when applying for federal grants or cooperative agreements. This number can be obtained by calling 1-866-705-5711 or online at http://www.dnb.com/us/. The D&B number should be entered on line 11 of the face page of the PHS 398 form.

Letters of intent must be received by 25 January 2005, with 25 February 2005 the deadline for applications. The complete version of this announcement is available online at http://grants.nih.gov/grants/guide/rfa-files/RFA-GM-05-010.html#PartI.

Contact: James J. Anderson, Center for Bioinformatics and Computational Biology, NIGMS, 45 Center Dr, Rm 2As.25A, MSC 6200, Bethesda, MD 20892-6200 USA, 301-594-0943, fax: 301-480-2228, e-mail: andersoj@nigms.nih.gov; Jiayin (Jerry) Li, Center for Bioinformatics and Computational Biology, NIGMS, 45 Center Dr, Rm 2As.19F, MSC 6200, Bethesda, MD 20892-6200 USA, 301-594-0682, fax: 301-480-2004, e-mail: lij@nigms.nih.gov. Reference: RFA No. RFA-GM-03-009

